# Commentary on the VERB™ Campaign — Perspectives on Social Marketing to Encourage Physical Activity Among Youth

**Published:** 2004-06-15

**Authors:** Adrian Bauman

**Affiliations:** Center for Physical Activity and Health

The VERB™ campaign is a serious public health investment that aims to tackle the societal and health problems of inactivity and increasing obesity among young Americans (1,2). Worrisome trends in risk factors among young people throughout the developed world reflect the lack of clearly effective public health approaches. Effecting population-level change is difficult, given the ingrained societal acceptability of sedentary behaviors and over-nutrition. VERB is an innovative and expansive effort to improve the current state of affairs, commencing with a national paid mass media campaign designed to reframe beliefs and norms about being active among *tweens* — children aged nine to 13 years. Secondary campaign objectives are to identify and influence key stakeholders, such as parents and teachers, and to work within communities to support opportunities for youth physical activity (1).

A campaign to influence physical activity should focus first on affecting social norms (3). Short-term goals should include documentation of changes in proximal variables (i.e., awareness, beliefs, and attitudes). But media alone cannot change behavior, because it provides only a preliminary cue for action. Behavior change should be the long-term goal of a sustained campaign. Long-term change is likely to take place only after translating and disseminating programs developed to support the mass communication components (3,4).

Previous youth media campaigns have targeted tobacco use, illicit drugs, and sexual health (5-7). These campaigns have had some success in increasing awareness of an issue, changing social norms toward substance use or the risk of sexually transmitted diseases, and offering solutions for young people to prevent tobacco uptake, call or ask for help in reducing drug use, or practice safe sexual behavior (8). VERB is the first substantial youth campaign, however, to increase youth activity and encourage a healthy lifestyle. VERB targets proximal outcomes, such as beliefs about inactivity, and encourages tweens to “find their verbs” —activities they might try and enjoy. VERB promotes the notion that not only can activity be enjoyable but it also can foster friendships with peers, enhance curiosity, and generate positive feelings of autonomy. Creating and maintaining these values are essential prerequisites to adopting and maintaining physical activity throughout adolescence.

VERB is highly intense for a public sector campaign, but it remains modest amid the plethora of marketing messages targeting tweens. Public health campaigns that use paid media messages, including campaigns that promote physical activity, are often reported outside the United States (9-11), but within the United States, the costs of paid media generally prohibit their use for public health messages, and public service announcements (PSAs) are instead typically used. Although local media campaigns might rely on PSAs for effect or on local-level media, which is less expensive (12), national initiatives require a much greater investment to achieve recognition. Any amount invested, however, remains miniscule compared to the health and social costs of inactivity and obesity, or indeed to the amount spent on commercial marketing to tweens. Thus, VERB represents a strong commitment to improving youth health because it requires a large investment in paid media.

The public health challenge is to penetrate the commercial-marketing media morass with well-designed messages that reach their target population. Inducing change in beliefs and norms is only the first step, however. Subsequent challenges are to create physical environments and spaces for tweens to move, play, and be active. The challenge involves advocacy, support, and policy change at the local and state levels to provide resources to construct or redevelop activity-friendly environments, such as schools, parks, trails, and neighborhoods. VERB extends beyond a media campaign and emphasizes the need to form community partnerships and coalitions to reinforce the media component and initiate community events (1). Community commitment poses the greatest challenge: VERB sustainability will be determined not only by continued efforts to influence youth beliefs but also by persuading decision makers to deploy long-term resources at the community level.

VERB employs elements of a social marketing framework: it applies marketing techniques, including promotional strategies utilizing place (i.e., multiple channels and venues), with a clearly defined and branded *product* (i.e., encouraging youths to find their “verbs”) (1). Consistent with any social marketing effort (13), VERB proposes a voluntary exchange: tweens who take up activity, presumably in place of watching television or just sitting around, will derive the benefits of fun and social engagement. VERB clearly segments its audience; although mainstream VERB messages target all tweens, ethno-specific VERB messages target minority youth. If long-term sustainability of VERB is to be ensured, the initiative has the potential to develop into a formal social marketing campaign, which would require implementation of the environmental, policy, and regulatory supports suggested as essential elements of effective social marketing (13).

Comprehensive evaluation is an essential component of a mass media campaign. The first stages of evaluation include understanding the needs and motivations of the target audience and developing clear messages for them (4,14). This process results in a defined brand that is recognizable, seen across different initiatives, and deemed relevant by the target group. VERB evaluation commenced with a logic model to provide a conceptual framework for the intervention (2). Most importantly, VERB carried out substantial formative evaluation to develop relevant and acceptable messages for tweens (http://www.cdc.gov/youthcampaign/research/formative.htm). Often neglected in campaign development, formative research helps in producing messages and brands more likely to be acted upon by the target population. Then, evaluators seek short-term impact on campaign awareness, beliefs about being active, and social norms among tweens, while looking for long-term impact on physical activity behavior (2). VERB assesses these proximal and explanatory variables, as well as physical activity itself. Multiple measures of reported physical activity are required to overcome the methodological problems of self-report or parental report of physical activity in this age group. Campaign literature seldom explores dose-response relationships, but VERB developed high-dose media communities and compares their results with those of standard-dose communities.

The prevention of chronic disease cannot be modeled in a causal relationship to youth media campaigns, because the reduced risk of chronic conditions may not appear for decades. We can consider the VERB initiative a public health policy success if trends in childhood inactivity and obesity are reversed within a decade, consistent with *Healthy People 2010* objectives. VERB-commissioned population surveys track proximal impact data; longer-term monitoring could occur through routine youth health surveys such as the Centers for Disease Control and Prevention’s Youth Risk Behavior Surveillance System (15). Process evaluation determines levels of VERB uptake by communities and minority populations in addition to measuring its impact on changing local policies and developing supportive community partnerships and sustainable physical environments.

Increases in rates of childhood obesity are not new, and declines in physical activity during adolescence are also well recognized in the scientific literature. Hence, it is timely that VERB was developed in an attempt to tackle these problems. VERB campaign efforts are not the end of the process but merely a well-resourced beginning upon which other efforts should build, synergize, and extend in partnership with community and state agencies to achieve population-level change. At the start of any such initiative, large-scale investment may be required as the spark plug to catalyze the first steps towards more active, healthier teenagers who have “found their verbs.”
